# Supplementary figures and images for: Demonstration of Patient-Specific Simulations to Assess Left Atrial Appendage Thrombogenesis Risk
Source: Front Physiol. 2021 Feb 26;12:596596. doi: 10.3389/fphys.2021.596596 (PMC7953154; doi:10.3389/fphys.2021.596596)

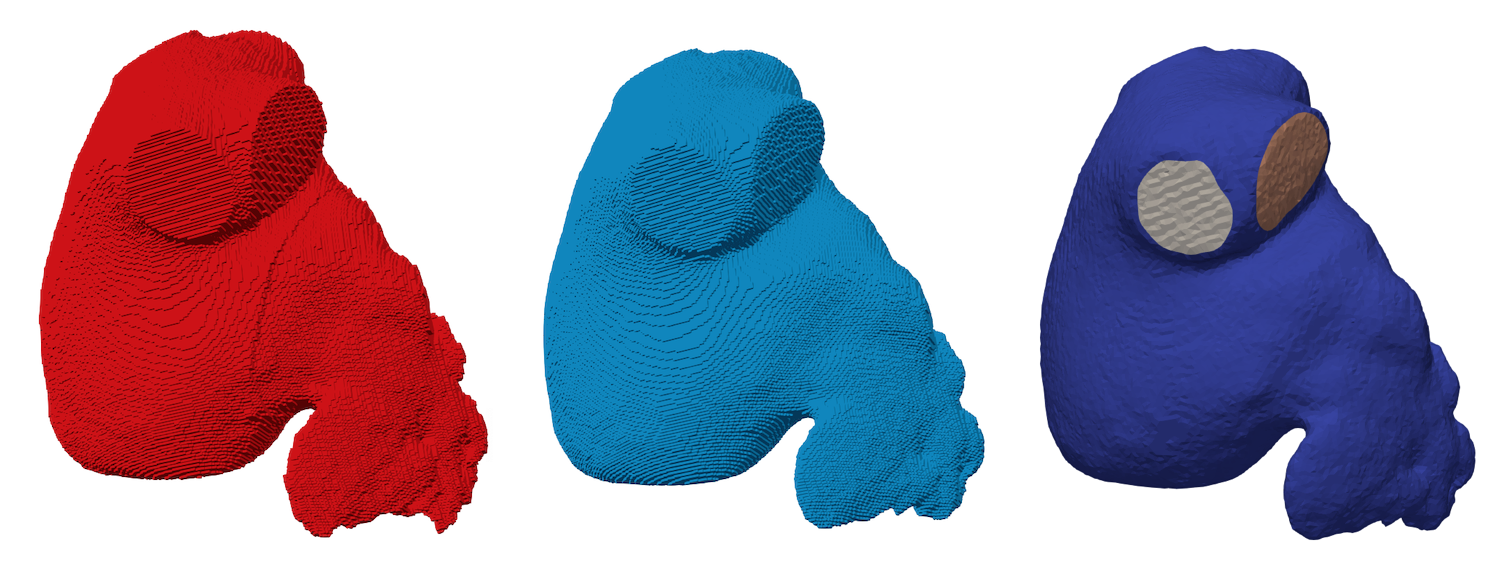

Supplement: Supplementary file 1 [file Image_1.TIFF]

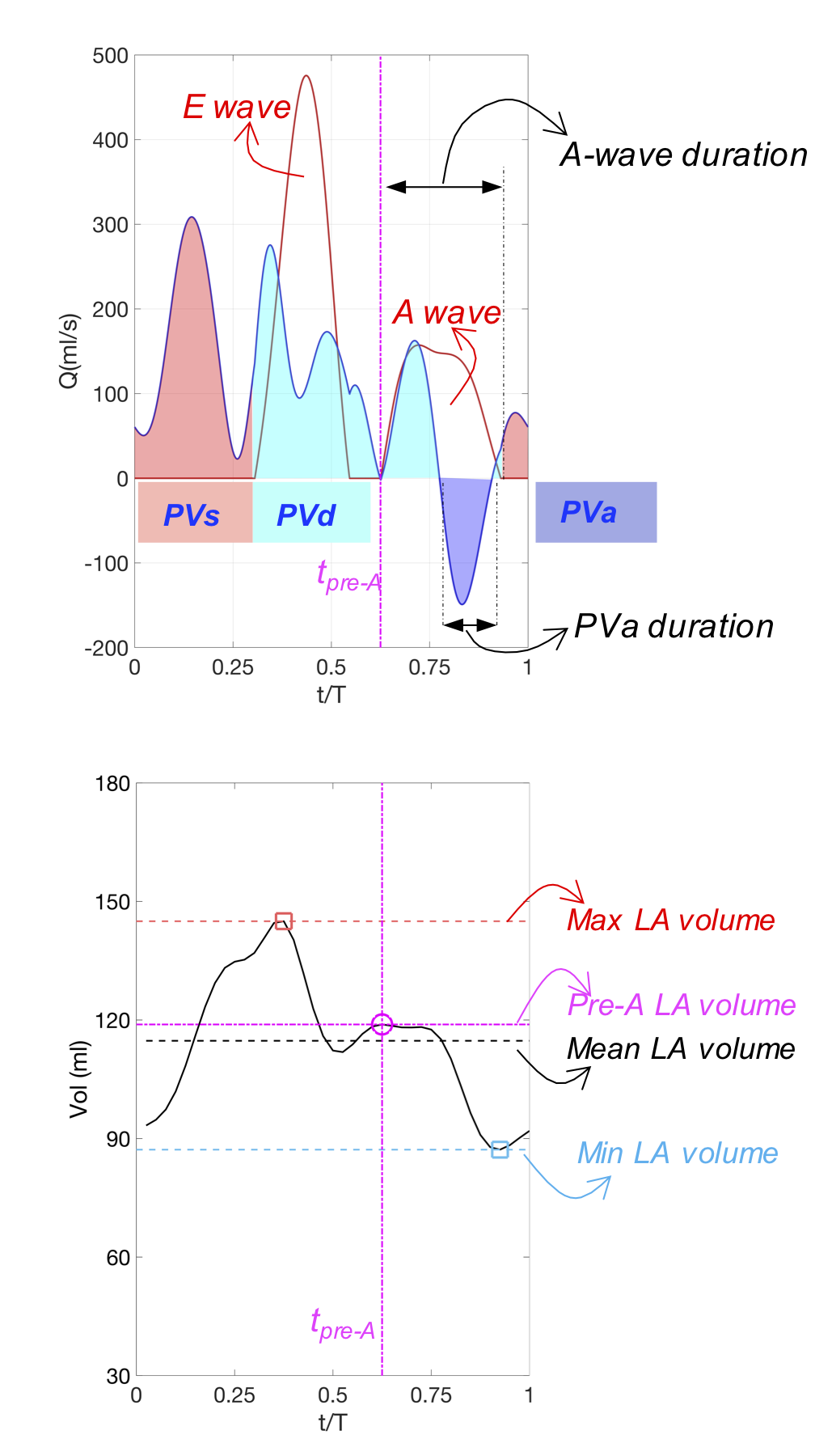

Supplement: Supplementary file 2 [file Image_2.TIFF]
