# Supplementary material for: Demonstration of Patient-Specific Simulations to Assess Left Atrial Appendage Thrombogenesis Risk
Source: Front Physiol. 2021 Feb 26;12:596596. doi: 10.3389/fphys.2021.596596 (PMC7953154; doi:10.3389/fphys.2021.596596)
Supplement: Supplementary file 3 [file Data_Sheet_1.docx]

Supplementary Material

## Supplementary Figures

**Supplementary Figure 1.** Illustration of image processing for one time frame corresponding to a subject with normal atrial function. **A)** Volume segmentation obtained after processing with itkSNAP. **B)** Same volume after filtering (smoothing and regularization). **C)** Final grid after the triangulation and registration process.

**Supplementary Figure 2.** Illustration of the calculation of LA functional parameters shown in Table 1 in a subject with normal atrial function. The **top panel** shows time histories of the flow rate through the mitral valve (**red**) and the cumulative flow rate through the pulmonary veins **(blue),** similar to Figures 3-5. These data are utilized to calculate: *PVs* (blood volume that enters the LA during LV systole, **red shadowed area**), *PVd* (blood volume that enters the LA during LV diastole, **light blue shadowed area**), *PVa* (blood volume that exits the LA due to reverse flow volume through the pulmonary veins during atrial contraction, **dark blue shadowed area**), *E/A* ratio (ratio of peak mitral velocities during early diastole and atrial contraction), and the instant of A-wave onset (*t_pre-A_***, magenta**). The **bottom panel** shows the LA volume vs. time, where maximum, minimum, mean and pre-LA values are indicated.
